# Oat Beta-Glucan Alone and in Combination with Hydrochlorothiazide Lowers High Blood Pressure in Male but Not Female Spontaneously Hypertensive Rats

**DOI:** 10.3390/nu15143180

**Published:** 2023-07-18

**Authors:** Pema Raj, Karen Sayfee, Liping Yu, Ali Sabra, Champa Wijekoon, Lovemore Malunga, Sijo Joseph Thandapilly, Thomas Netticadan

**Affiliations:** 1 St. Boniface Hospital Research Centre, Winnipeg, MB R2H 2A6, Canada; praj@sbrc.ca (P.R.); ali.sabra@agr.gc.ca (A.S.);; 2 Canadian Centre for Agri-Food Research in Health and Medicine, Winnipeg, MB R2H 2A6, Canada; 3 Agriculture and Agri-Food Canada, Winnipeg, MB R2H 2A6, Canada; lovemore.malunga@agr.gc.ca; 4 Department of Human Nutritional Sciences, University of Manitoba, Winnipeg, MB R2H 2A6, Canada; 5 Richardson Center for Food Technology and Research, Winnipeg, MB R2H 2A6, Canada; 6 Department of Physiology and Pathophysiology, University of Manitoba, Winnipeg, MB R2H 2A6, Canada

**Keywords:** beta-glucan, hydrochlorothiazide, hypertension, cardiac function, oxidative stress

## Abstract

Oats are considered a functional food due to the beneficial health effects associated with their consumption and are suitable to be explored for their ability to prevent or manage chronic disease, such as hypertension. Here, we examined the cardiovascular benefits of an oat beta-glucan extract in male and female spontaneously hypertensive rats (SHRs) to unravel its sex-specific roles when used with an anti-hypertensive medication, hydrochlorothiazide. Five-week-old male and female SHRs and Wistar–Kyoto (WKY) rats were treated with oat beta-glucan and hydrochlorothiazide for 15 weeks. Twenty-week-old male and female SHRs showed high blood pressure (BP), cardiac remodeling, and cardiac dysfunction. These animals also had significantly increased levels of malondialdehyde (MDA), angiotensin II, and norepinephrine. Treatments with beta-glucan and hydrochlorothiazide were able to differentially prevent high BP, cardiac dysfunction, and alterations in malondialdehyde (MDA), angiotensin II, and norepinephrine in 20-week-old male and female SHRs. To conclude, beta-glucan alone and in combination with hydrochlorothiazide may be a promising a strategy for managing hypertension and related cardiac complications.

## 1. Introduction

Hypertension is known to cause cardiovascular abnormalities, such as atherosclerosis, cardiac hypertrophy, myocardial infarction, cardiac arrest, heart failure, hemorrhagic stroke, peripheral artery disease, chronic kidney disease, and cognitive impairment [1,2]. Because hypertension contributes to the development of many complex diseases, it has been recognized as the most debilitating risk factor that affects the world population [1,2]. Moreover, hypertension is associated with the worsening of health-related quality of life, irrespective of other comorbidities [3]. Essential or primary hypertension is aetiologically heterogeneous and is influenced by genetics and living environment [3,4]. Systolic blood pressure (BP) levels tend to rise steadily and continuously with aging in both men and women. BP is a sexually dimorphic physiological phenotype, and the prevalence of this condition can vary significantly between males and females [5]. These differences arise from biologically (sex) and psychosocially (gender) mediated factors [5].

Dietary fiber intake may reduce the risk of cardiovascular disease. Health benefits associated with oat consumption are mainly attributed to beta-glucan, a soluble fiber present in oats [6]. Beta-glucan is a linear polysaccharide with monomers of glucose with beta (1–4) and beta (1–3) linkages, and its concentrations typically range from 3.9% to 6.8% in oats [6]. Beta-glucans are known to lower blood glucose and cause cholesterol levels to circulate due to their physiochemical characteristics, such as molecular weight and viscosity [7,8,9]. They can reduce the absorption of dietary cholesterol, promote its excretion, and inhibit cholesterol synthesis in the liver. Beta-glucan may also have the potential to manage BP [10,11]; however, this aspect needs to be established unequivocally for researchers to be able to achieve a health claim for BP management. Furthermore, there is no evidence on whether beta-glucan has a sex-specific effect in lowering BP in males and females. Importantly, evidence-based BP management involves using agents such as diuretic hydrochlorothiazide in monotherapy or with combination treatments using angiotensin-converting enzyme inhibitors, calcium channel blockers, and beta-blockers that can lower BP. Hence, it is also imperative to understand the efficacy of beta-glucan alongside other therapeutic agents in a comparative and combinatorial manner.

The spontaneously hypertensive rat (SHR) is the most used animal model for exploring the mechanisms underlying the development and progression of primary hypertension, as well as for developing potential new strategies for preventing or treating primary hypertension. SHRs are known to develop significant cardiac abnormalities, similar to those observed in humans with hypertension. SHRs exhibit diastolic dysfunction, characterized by impaired relaxation and, thus, compliance of the cardiac chambers by 15 weeks of age. Although the individual effects of oat beta-glucan and hydrochlorothiazide are documented, the specific combinatorial effects of oat beta-glucan and hydrochlorothiazide are not known. In addition, the different impacts of these two compounds in males vs. females has not been assessed. Therefore, we hypothesized that oat beta-glucan alone and in a combination with hydrochlorothiazide would lower BP and prevent cardiac abnormalities in male and female SHRs. In the current study, we investigated the potential of oat beta-glucan both individually and in a combination with hydrochlorothiazide for preventing hypertension and cardiovascular abnormalities in male and female SHRs.

## 2. Materials and Methods

### 2.1. Animal Ethics

All animal experiments performed in this study were sanctioned by the University of Manitoba Animal Care Committee (Approval Code: AC11291; Approval Date: 24 August 2018) and were performed as per the recommendations of the Canadian Council on Animal Care and Use of Experimental Animals. Four-week-old male and female SHRs and Wistar–Kyoto (WKY) rats were procured from Charles River, Inc., Montreal, QC, Canada. Animals were acclimatized for 1 week in the animal care facility in temperature- and humidity-controlled rooms with a 12 h dark and 12 h light period cycle and kept in the same conditions for the rest of the study period.

### 2.2. Animal Model of Hypertension and Study Treatment Regimen

SHRs were used in this study as the animal model of essential hypertension. WKY rats served as normotensive controls. The occurrence and nature of cardiac hypertrophy in SHRs resembles the development of hypertrophy secondary to essential hypertension in humans [12,13]. More importantly, SHRs develop hypertrophy and heart failure gradually in response to progressive hypertensive disease, not as a consequence of a surgical procedure (surgical models) or chemicals (NG-Nitroarginine methyl ester, N-Nitro-L-arginine methylester). The SHR strain was obtained during the 1960s by Okamoto and colleagues via the selective inbreeding of normal WKY rats that developed hypertension, and this is the reason why WKY rats have been used as the normotensive control in all previous studies. In SHRs, hypertension begins around 6 weeks of age. From 2 months of age, SHRs develop heart dysfunction. The stage of heart failure occurs from approximately 18 months of age [12,13]. Five-week-old male SHRs and their age-matched controls, WKY rats, were employed in this study. WKY rats and SHRs were treated daily with oral gavage for a period of 15 weeks with a vehicle (deionized water), oat derived beta-glucan (305 mg/kg/day), or a combination of oat beta-glucan (305 mg/kg/day) and hydrochlorothiazide (20 mg/kg/day). Gavage began when the rats reached 5 weeks of age and continued daily until the rats reached 20 weeks of age. The oat beta-glucan (OGLUCAN70, China) was bought from Garuda International Inc., San Francisco, CA, USA, and had an average molecular weight of 73,759 g/mol and purity of 83% *w*/*w* db. Animals were sacrificed and blood (serum was separated) and heart tissue samples were collected to the study the effects on heart and serum biochemical parameters.

### 2.3. Transthoracic Echocardiography

Cardiac structure and function were assessed in all groups of animals by performing transthoracic echocardiography at 15 weeks of treatment. Transthoracic two-dimensional (2D) guided M-mode and pulse-wave Doppler measurements were performed using an ultrasound system (VIVID E9, Boston, MA, USA) equipped with a 13 MHz transducer suitable for rodents, as described by us earlier [14,15]. The images for each measurement were recorded for at least 3 cardiac cycles. The EchoPac software (Boston, MA, USA) was used for an analysis of the images. The 2D M-mode measurements included cardiac structural parameters, such as interventricular septal wall thickness (IVS), left ventricular posterior wall thickness (LVPW), and left ventricular internal dimensions (LVID) at diastole, and systolic functional parameters, such as the left ventricular ejection fraction (LVEF), cardiac output (CO), and stroke volume (SV). Doppler measurements included a diastolic functional parameter, the isovolumetric relaxation time (IVRT). Animals were anaesthetized while undergoing echocardiography.

### 2.4. BP Measurement

BP measurement was carried out in both male and female WKY rats and SHRs at 0 (baseline) and 15 weeks of treatment, as described previously [15]. A CODA multi-channel, computerized, non-invasive BP system (Kent Scientific, Torrington, WY, USA) with a tail-cuff sphygmomanometer was used to measure systolic and approximate diastolic BP in conscious rats. Briefly, during each measurement cycle, blood was pushed away from the tail by the volume pressure recording cuff. The occlusion cuff stopped the backflow of the blood into the tail. When the occlusion cuff deflated, it let the blood flow back into the tail and, thereby, increased the tail volume. The pressure exerted by the occlusion cuff during this increase in tail volume was measured as systolic BP, whereas the occlusion cuff pressure at which blood flows into and out of the tail and equalizes during deflation was defined as the diastolic pressure.

### 2.5. Serum Biochemical Assays

Oxidative stress was determined by assessing the levels of lipid peroxidation in the serums, LVs, and aortas of each group by measuring the amount of a lipid peroxidation product called malondialdehyde (MDA). This was performed using a thiobarbituric acid reactive substance-based assay kit (Abcam, Waltham, USA), as described previously, following the manufacturer’s instructions. The enzyme activity of antioxidant enzymes, such as super oxide dismutase (SOD) and catalase, were estimated in the serums using enzyme assay kits (Abcam, Waltham, MA, USA), as described previously, following the manufacturer’s instructions. The status of inflammation was determined by measuring the concentration of the inflammatory cytokine, and the tumor necrosis factor alpha (TNF-alpha) was determined using an ELISA assay kit (Abcam, Waltham, MA, USA), as described previously, following the manufacturer’s instructions. Nitric oxide is a potent vasodilator and an index of vascular function. NO is highly reactive and has a very short half-life. NO is converted to nitrite (NO^2−^) and nitrate (NO^3−^), and the total concentration of nitrite and nitrate can be used as a quantitative measure of NO production. Hence, nitrate (NO^3−^) and nitrite (NO^2−^) were measured in the serums from all groups of SHRs and WKY rats, as per the manufacturer’s instructions (Abcam, Waltham, USA). Neurohormones in the serums, such as angiotensin II (serum) and norepinephrine (serum), were also determined using the ELISA assay kit (Abcam, Waltham, USA). Cardiac fibrosis was determined by assessing the concentrations of hydroxy proline (LV) using a hydroxyproline assay. Gas chromatography–mass spectrometry was performed using a Bruker 436-GC equipped with an EVOQ-TQ-MS (Bruker Daltonics, Bremen, Germany) to detect short chain fatty acids in the serums [16].

### 2.6. Statistical Analysis

All values were expressed as means ± SEM. A one-way analysis of variance was used to analyze variations between the means of the groups in the study (Graphpad Prism, Boston, MA, USA). Significant values are defined as *p* < 0.05. When significance was obtained, the one-way analysis of variance was followed by a Newman–Keuls post hoc test.

## 3. Results

### 3.1. Body Weight and Heart/Tibia Length in SHRs and WKY

**Figure 1:**
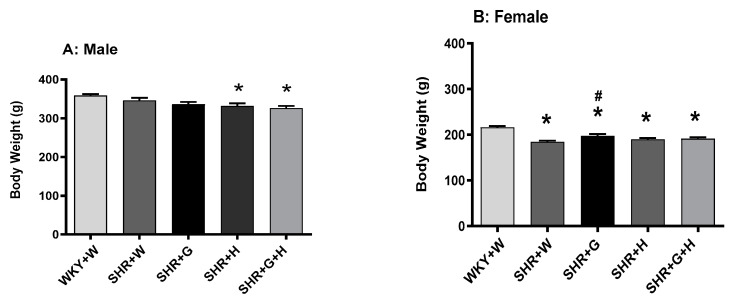
Effects of beta-glucan (G), hydrochlorothiazide (H), and beta-glucan + hydrochlorothiazide (G + H) on body weight of 20-week-old (**A**) male WKY rats and SHRs and (**B**) female WKY rats and SHRs. Values are means ± SEMs (n = 10 rats). Significant values are defined as *p* < 0.05. * *p* < 0.05 vs. WKY + W. # *p* < 0.05 vs. SHR + W.

**Figure 2:**
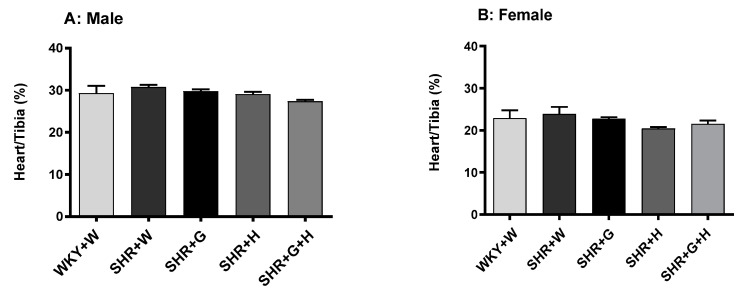
Effects of beta-glucan (G), hydrochlorothiazide (H), beta-glucan + hydrochlorothiazide (G + H) on LV-to-tibia length ratio after 15 weeks of treatment in (**A**) male WKY rats and SHRs and (**B**) female WKY rats and SHRs. Values are means ± SEMs (n = 10 rats).

Body weight was recorded weekly from the baseline to the endpoint of the study in both male and female WKY rats and SHRs. The body weight of male SHRs treated with hydrochlorothiazide and hydrochlorothiazide + beta-glucan (Figure 1A) was lower compared to that of male WKY rats. Although all female SHR groups had a significantly lower body weight than WKY rats (Figure 1B), beta-glucan consumption slightly improved body weight in female SHRs. In contrast, the heart-to-tibia length ratio (used as a surrogate marker of pathological cardiac hypertrophy) was not significantly different in any groups (Figure 2A,B), suggesting the absence of pathological remodeling.

**Figure 3:**
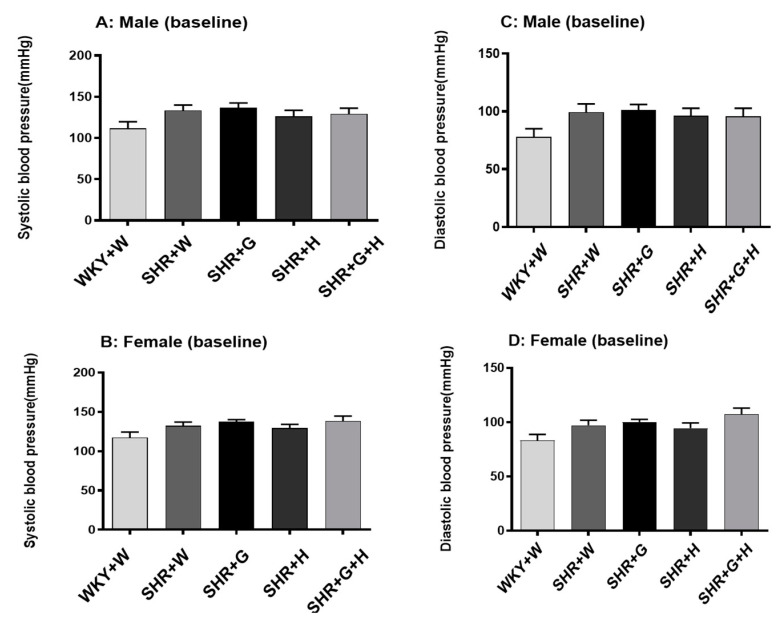
Effects of beta-glucan (G), hydrochlorothiazide (H), and beta-glucan + hydrochlorothiazide (G + H) on BP at baseline in five-week-old WKY rats and SHRs. (**A**) Systolic BP in male WKY rats and SHRs at baseline, (**B**) systolic BP in female WKY rats and SHRs at baseline, (**C**) diastolic BP in male WKY rats and SHRs, (**D**) diastolic BP in female WKY rats and SHRs. Values are means ± SEMs (n = 10 rats).

**Figure 4:**
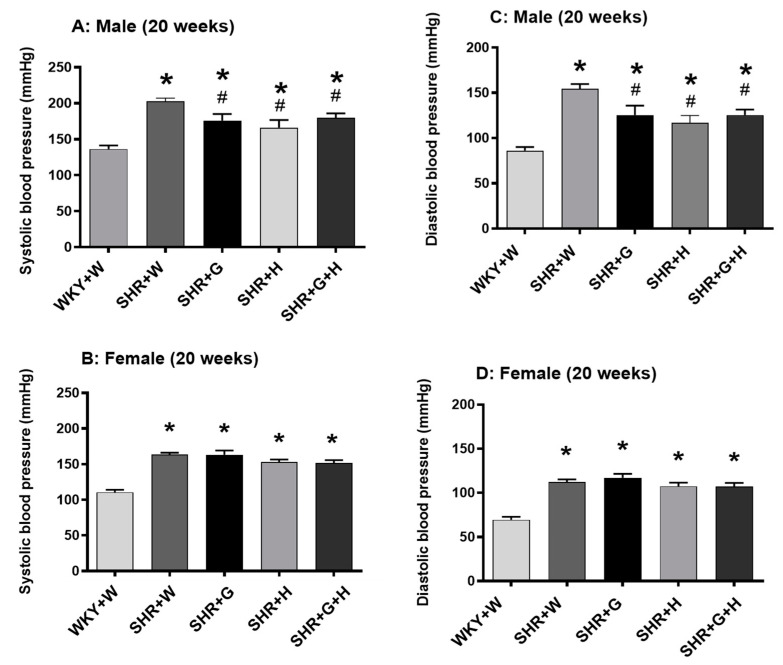
Effects of beta-glucan (G), hydrochlorothiazide (H), and beta-glucan + hydrochlorothiazide (G + H) on BP in 20-week-old WKY rats and SHRs. (**A**) Systolic BP in male WKY rats and SHRs, (**B**) systolic BP in female WKY rats and SHRs, (**C**) diastolic BP in male WKY rats and SHRs, (**D**) diastolic BP in female WKY rats and SHRs. Values are means ± SEMs (n = 10 rats). Significant values are defined as *p* < 0.05. * *p* < 0.05 vs. WKY + W. # *p* < 0.05 vs. SHR + W.

BP was measured before the start of treatment at baseline (week zero—all animals were five weeks of age) and at 20 weeks of age to determine sequential changes in BP in SHRs compared to WKY rats and to delineate the effectiveness of the three different study intervention strategies. There were no significant differences in systolic and diastolic BPs between male and female SHRs and WKY rats at baseline (Figure 3). Systolic and diastolic BPs were significantly elevated in 20-week-old male and female SHRs that received the vehicle when compared to age-matched male and female WKY rats that received the vehicle (Figure 4). Beta-glucan or hydrochlorothiazide alone resulted in significantly lowering systolic and diastolic BPs in male SHRs when compared to vehicle-treated male SHRs (Figure 4A,C). The combination treatment with beta-glucan and hydrochlorothiazide also resulted in lower systolic and diastolic BPs in male SHRs when compared to vehicle-treated male SHRs (Figure 4A,C). However, neither beta-glucan or hydrochlorothiazide alone nor a combination of both significantly reduced systolic and diastolic BPs in female SHRs when compared to vehicle-treated female SHRs (no blood pressure lowering effects) (Figure 4B,D).

### 3.2. Heart Structure and Function in WKY Rats and SHRs

**Figure 5:**
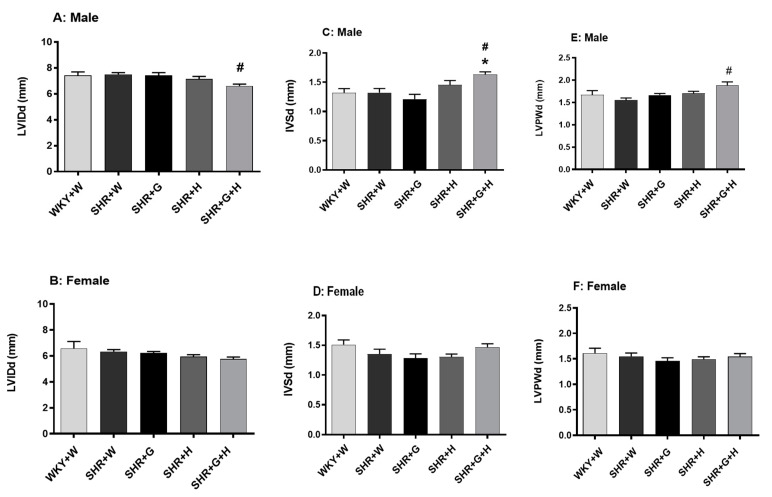
Effects of beta-glucan (G), hydrochlorothiazide (H), and beta-glucan + hydrochlorothiazide (G + H) on the cardiac structure of 20-week-old male and female WKY rats and SHRs after 15 weeks of treatment. (**A**,**B**) Left ventricular internal dimension (LVID) at diastole in male and female WKY rats and SHRs. (**C**,**D**) Interventricular septal wall thickness (IVS) at diastole in male and female WKY rats and SHRs. (**E**,**F**) Left ventricular posterior wall thickness (LVPW) at diastole in male and female WKY rats and SHRs. Values are means ± SEMs (n = 10 rats). Significant values are defined as *p* < 0.05. * *p* < 0.05 vs. WKY + W. # *p* < 0.05 vs. SHR + W.

The transthoracic echocardiography analysis showed that 20-week-old male and female SHRs treated with the vehicle did not exhibit any change in the internal dimensions of their heart chambers, such as that of the left ventricle (LVID), when compared to age-matched male and female WKY rats treated with the vehicle (Figure 5A,B), suggesting that there was no significant pathological cardiac dilation. Other heart chamber structural parameters, such as cardiac wall thicknesses, IVS, and LVPW, were also not altered between the groups in 20-week-old animals (Figure 5C–F). However, LVID, IVS, and LVPW were found to be significantly higher in male SHRs treated with beta-glucan and hydrochlorothiazide at diastole when compared to the control, WKY rats (Figure 5A,C,E).

**Figure 6:**
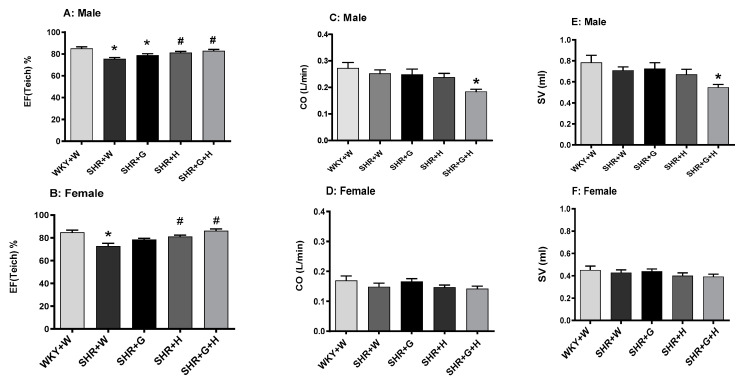
Effects of beta-glucan (G), hydrochlorothiazide (H), and beta-glucan + hydrochlorothiazide (G + H) on the cardiac function of 20-week-old male and female WKY rats and SHRs after 15 weeks of treatment. (**A**,**B**) Left ventricular ejection fraction (LVEF) in male and female WKY rats and SHRs, (**C**,**D**) cardiac output (CO) in male and female WKY rats and SHRs, and (**E**,**F**) stroke volume (SV) in male and female WKY rats and SHRs. Values are means ± SEMs (n = 10 rats). Significant values are defined as *p* < 0.05. * *p* < 0.05 vs. WKY + W. # *p* < 0.05 vs. SHR + W.

**Figure 7:**
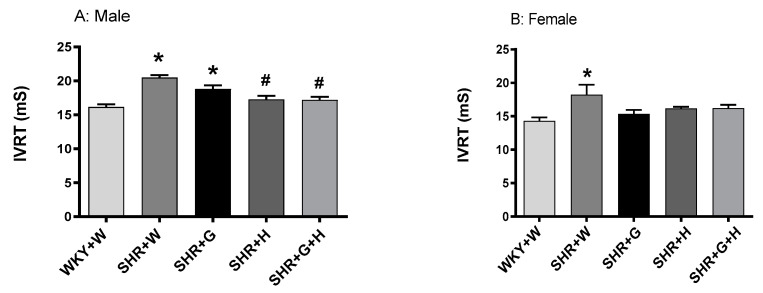
Effects of beta-glucan (G), hydrochlorothiazide (H), and beta-glucan + hydrochlorothiazide (G + H) on the isovolumic relaxation time (IVRT) of 20-week-old male and female WKY rats and SHRs after 15 weeks of treatment. (**A**) IVRT in male WKY rats and SHRs and (**B**) IVRT in female WKY rats and SHRs. Values are means ± SEMs (n = 10 rats). Significant values are defined as *p* < 0.05. * *p* < 0.05 vs. WKY + W. # *p* < 0.05 vs. SHR + W.

With regards to their heart pumping function, there was a significant reduction in systolic functional parameters, such as the LVEF, in 20-week-old vehicle-treated male and female SHRs in comparison to those in vehicle-treated male and female WKY rats (Figure 6A,B). Male and female SHRs treated with hydrochlorothiazide or the combination of beta-glucan and hydrochlorothiazide had a significantly higher LVEF compared to that of vehicle-treated male and female SHRs (Figure 6A,B), suggesting an improvement in their heart pumping function. CO and SV were comparable between 20-week-old male and female WKY rats and SHRs (Figure 6C–F). Male SHRs treated with the combination had lower CO and SV when compared to control WKY rats (Figure 6C,E). Diastolic functional parameters, such as the IVRT, were significantly higher in 20-week-old vehicle-treated male and female SHRs in comparison to those of WKY rats treated with the vehicle (Figure 7A,B), suggesting an impairment in the relaxation of heart muscle. Male SHRs treated with hydrochlorothiazide and the combination of beta-glucan and hydrochlorothiazide had significantly lower IVRTs compared to vehicle-treated male SHRs (Figure 7A).

### 3.3. Biochemical Parameters in WKY Rats and SHRs

**Figure 8:**
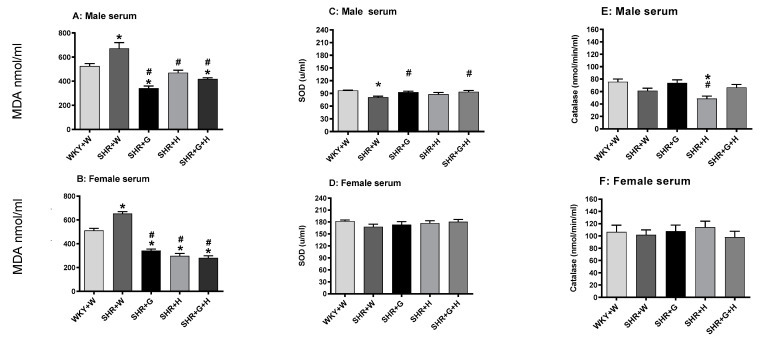
Effects of beta-glucan (G), hydrochlorothiazide (H), and beta-glucan + hydrochlorothiazide (G + H) on serum malondialdehyde (MDA), super oxide dismutase (SOD), and catalase in 20-week-old male and female WKY rats and SHRs. (**A**) Serum MDA in male WKY rats and SHRs and (**B**) Serum MDA in female WKY rats and SHRs. (**C**) Serum SOD in male WKY rats and SHRs and (**D**) Serum SOD in female WKY rats and SHRs. (**E**) Serum catalase in male WKY rats and SHRs and (**F**) Serum catalase in female WKY rats and SHRs Values are means ± SEMs (n = 6 rats). Significant values are defined as *p* < 0.05. * *p* < 0.05 vs. WKY + W. # *p* < 0.05 vs. SHR + W.

**Figure 9:**
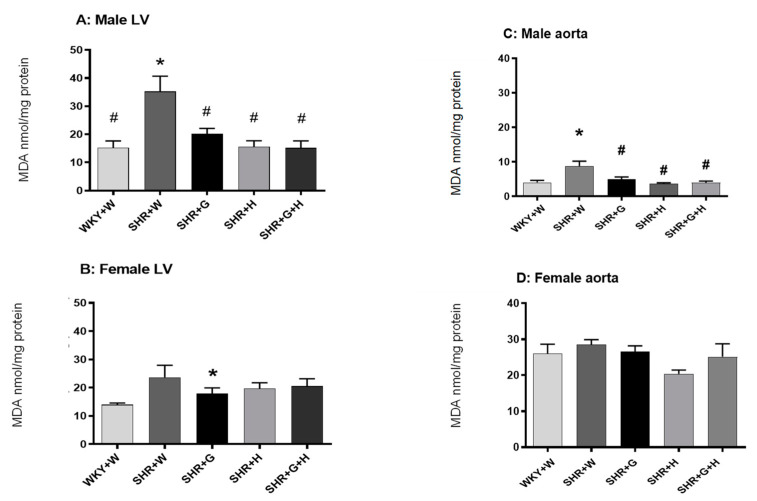
Effects of beta-glucan (G), hydrochlorothiazide (H), and beta-glucan + hydrochlorothiazide (G + H) on LV and aortic malondialdehyde (MDA) in 20-week-old male and female WKY rats and SHRs. (**A**) MDA in the LVs of male WKY rats and SHRs and (**B**) MDA in the LVs of female WKY rats and SHRs. (**C**) MDA in the aortas of male WKY rats and SHRs and (**D**) MDA in the aortas of female WKY rats and SHRs. Values are means ± SEMs (n = 6 rats). Significant values are defined as *p* < 0.05. * *p* < 0.05 vs. WKY + W. # *p* < 0.05 vs. SHR + W.

Twenty-week-old male and female SHRs treated with the vehicle had significantly higher levels of MDA in their serums in comparison to those in the serums of male and female WKY rats treated with the vehicle (Figure 8A,B), signifying the presence of oxidative stress. Beta-glucan, hydrochlorothiazide, and the combination treatments significantly reduced levels of MDA in the serums of male and female SHRs in comparison to those in the serums of vehicle-treated SHRs (Figure 8A,B). SOD activity in vehicle-treated 20-week-old male SHRs was significantly lower than that in age-matched WKY rats (Figure 8C). Male SHRs treated with beta-glucan or the combination of beta-glucan and hydrochlorothiazide had higher levels of SOD activity compared to those of vehicle-treated male SHRs (Figure 8C). There were no alterations in SOD activity between 20-week-old female WKY rats and the SHR groups (Figure 8D). Serum catalase activity was not different between twenty-week-old male and female vehicle-treated SHRs and WKY rats (Figure 8E,F); however, there was a significant decrease in the levels of this enzyme in SHRs treated with hydrochlorothiazide (Figure 9E). Twenty-week-old male SHRs treated with the vehicle also had significantly higher levels of MDA in their LVs and aortas than did age-matched male WKY rats, whereas 20-week-old female WKY rats and SHRs treated with the vehicle had comparable levels of MDA in their LVs and aortas (Figure 9A–D). All treatments were able to lower MDA levels in the LVs and aortas of male SHRs (Figure 9A,C).

**Figure 10:**
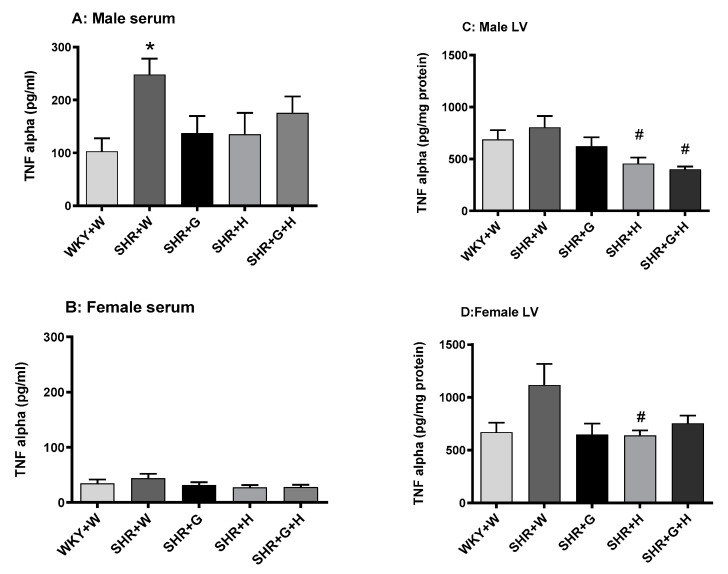
Effects of beta-glucan (G), hydrochlorothiazide (H), beta-glucan + hydrochlorothiazide (G + H) on the tumor necrosis factor-alpha (TNF-alpha) in 20-week-old male and female WKY rats and SHRs. (**A**) Serum TNF-alpha in male WKY rats and SHRs and (**B**) serum TNF-alpha in female WKY rats and SHRs. (**C**) TNF-alpha in the LV of male WKY rats and SHRs and (**D**) TNF-alpha in the LV of female WKY rats and SHRs. Values are means ± SEMs (n = 6 rats). Significant values are defined as *p* < 0.05. * *p* < 0.05 vs. WKY + W. # *p* < 0.05 vs. SHR + W.

**Figure 11:**
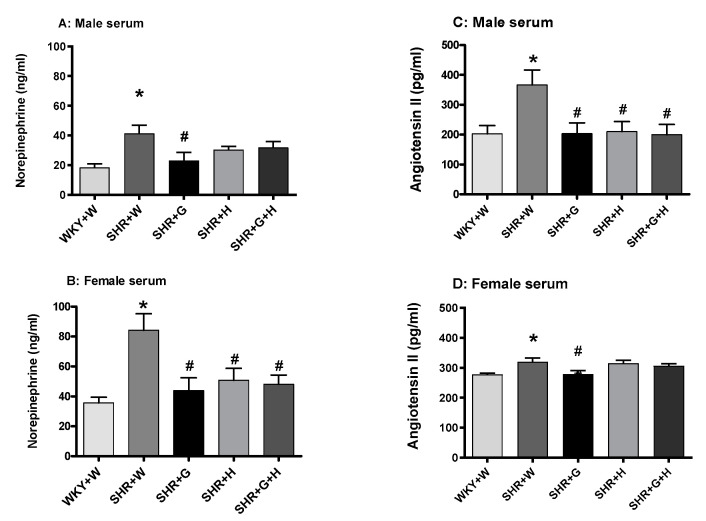
Effects of beta-glucan (G), hydrochlorothiazide (H), and beta-glucan + hydrochlorothiazide (G + H) on norepinephrine and angiotensin II in 20-week-old male and female WKY rats and SHRs. (**A**) Serum norepinephrine in male WKY rats and SHRs and (**B**) serum norepinephrine in female WKY rats and SHRs. (**C**) Serum angiotensin II in male WKY rats and SHRs and (**D**) serum angiotensin II in in female WKY rats and SHRs. Values are means ± SEMs (n = 6 rats). Significant values are defined as *p* < 0.05. * *p* < 0.05 vs. WKY + W. # *p* < 0.05 vs. SHR + W.

Serum levels of TNF-alpha in vehicle-treated, 20-week-old male SHRs were significantly higher than those in male WKY rats (Figure 10A), suggesting the presence of inflammation, whereas LV levels of TNF-alpha in male WKY rats and SHRs were comparable (Figure 10C). Serum and LV levels of TNF-alpha in vehicle-treated, 20-week-old female SHRs and WKY rats were also not significantly different (Figure 10B,D). Twenty-week-old male and female SHRs treated with the vehicle had significantly higher levels of norepinephrine in their serums than did male and female WKY rats treated with the vehicle (Figure 11A,B). Beta-glucan, hydrochlorothiazide, and the combination treatments significantly lowered levels of norepinephrine in female SHRs in comparison to those of vehicle-treated SHRs (Figure 11B), whereas beta-glucan-treated male SHRs had lower levels of norepinephrine than did vehicle-treated SHRs (Figure 11A).

**Figure 12:**
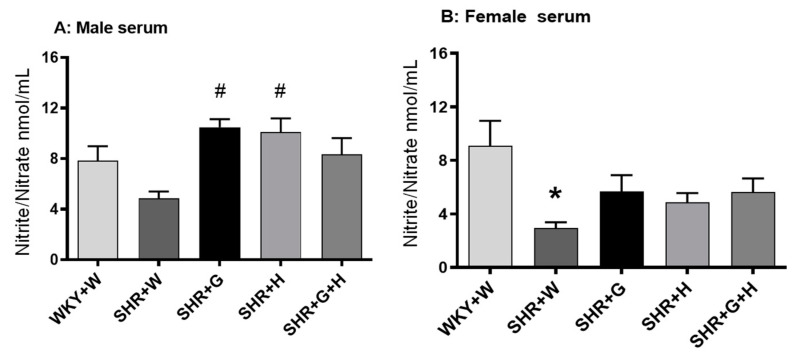
Effects of beta-glucan (G), hydrochlorothiazide (H), and beta-glucan + hydrochlorothiazide (G + H) on nitrite (NO2-)/nitrate (NO3-) in 20-week-old WKY rats and SHRs after 15 weeks of treatment. (**A**) Serum nitrite/nitrate in male WKY rats and SHRs. (**B**) Serum nitrite/nitrate in female WKY rats and SHRs. Values are means ± SEMs (n = 6 rats). Significant values are defined as *p* < 0.05. * *p* < 0.05 vs. WKY + W. # *p* < 0.05 vs. SHR + W.

**Figure 13:**
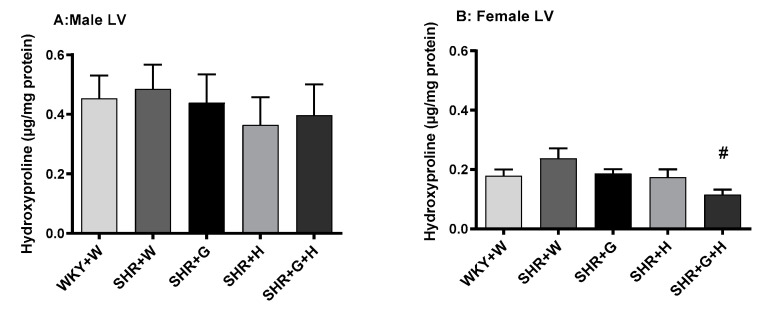
Effects of beta-glucan (G), hydrochlorothiazide (H), and beta-glucan + hydrochlorothiazide (G + H) on hydroxyproline in 20-week-old WKY rats and SHRs after 15 weeks of treatment. (**A**) Hydroxyproline in the LVs of male WKY rats and SHRs. (**B**) Hydroxyproline in the LVs of female WKY rats and SHRs. Values are means ± SEMs (n = 6 rats). Significant values are defined as *p* < 0.05. # *p* < 0.05 vs. SHR + W.

We also observed that serum levels of angiotensin II in 20-week-old male and female SHRs treated with the vehicle were higher than those in age matched male and female WKY rats treated with the vehicle (Figure 11C,D). Treatments with beta-glucan, hydrochlorothiazide, or the combination of both resulted in significantly lower levels of angiotensin II in male SHRs than in vehicle-treated SHRs (Figure 11C), whereas beta-glucan-treated female SHRs had lower levels of angiotensin II (Figure 11D). The serum levels of nitric oxide (nitrate/nitrite) were not significantly different between 20-week-old male WKY rats and SHRs (Figure 12A). However, there was a trend towards lower levels of NO in SHRs than in WKY rats (Figure 9A). The serum levels of nitric oxide (measured as nitrate/nitrite) were significantly lower in vehicle-treated female SHRs than in vehicle-treated female WKY rats (Figure 12B). The levels of nitric oxide (nitrate/nitrite) were not significantly different between treated and untreated female SHRs (Figure 12B). LV hydroxy proline levels were not significantly different between 20-week-old male and female WKY rats and male and female SHRs (Figure 13A,B)).

**Figure 14:**
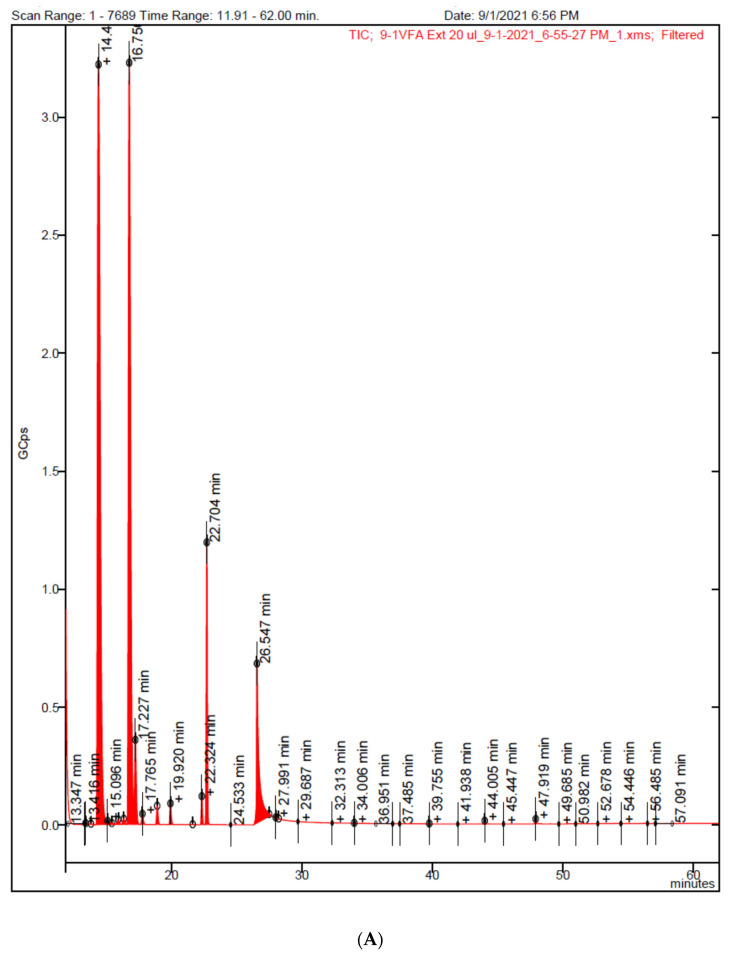

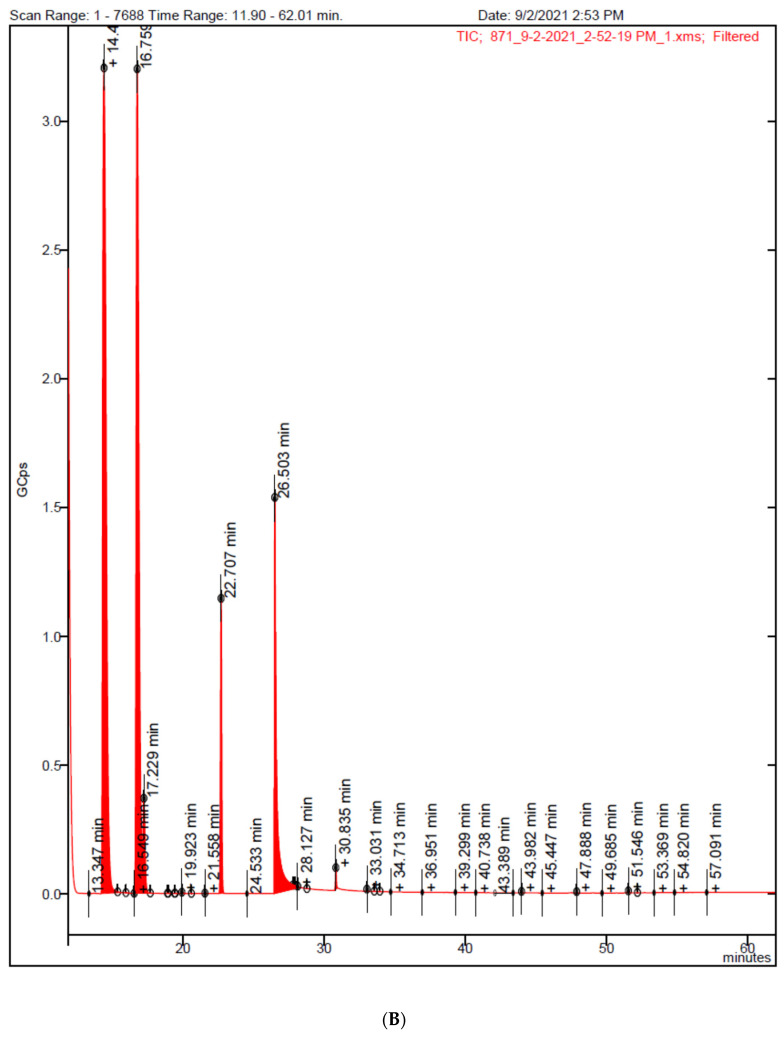
(**A**) GCMS chromatogram of a mix of SCFA standards that were extracted using the same method used for the serum samples. Rt 13.416 formic acid butyl ester; 13.825 acetic acid butyl ester; 14.8 propionic acid butyl ester; 15.909 butyric acid butyl ester; 16.338 isovaleric acid butyl ester; 17.227 butyl-2-ethylbutyrate (internal standard); 17.765 valeric acid butyl ester; 18.903 isohexanoic acid butyl ester; 19.920 hexanoic acid butyl ester; 22.324 heptanoic acid butyl ester. (**B**) GCMS chromatogram of serum sample indicating no SCFA was detected. Major peaks in both chromatograms are solvents at Rt. 14.4 min butyl alcohol; Rt 16.7 min pyridine; and Rt 22.7 min carbonic acid dibutyl ester.

The analysis of beta-glucan derived short-chain fatty acids in the serums revealed that the level was below the detection limit for all groups of animal samples tested (Figure 14), suggesting that there is no role for short-chain fatty acids in beta-glucan-mediated blood pressure lowering.

## 4. Discussion

This study reports, for the first time, the different sex-specific effects of beta-glucan alone and in combination with the anti-hypertensive medication hydrochlorothiazide on hypertension and cardiac structural and functional abnormalities in male and female SHRs. We found that beta-glucan treatment for 15 weeks in young male SHRs had a significant anti-hypertensive effect. Of note, this anti-hypertensive effect brought about by beta-glucan was comparable to hydrochlorothiazide, which has a potent BP lowering efficacy. Beta-glucan alone and in combination with hydrochlorothiazide also improved systolic function in both 20-week-old male and female SHRs.

At five weeks of age, both male and female SHRs and WKY rats exhibited no significant difference in their systolic and diastolic BPs, suggesting that hypertension developed post five weeks of age in the SHRs in this study. By 20 weeks of age, both male and female vehicle-treated SHRs had very high BP compared to those of age- and sex-matched male and female WKY rats, as reported by earlier literature. Male SHRs had higher BP compared to female SHRs at 20 weeks of age. This observation is consistent with the previous literature showing that male SHRs have higher BP than their female counterparts [17,18]. Other models of hypertension, such as Dahl salt-sensitive hypertension and deoxy corticosterone sensitive hypertension, also exhibit sex-specific differences in BP [17,19]. In humans, men are known to have higher risks of hypertensive heart disease than age-matched pre-menopausal women [17,20]. However, after menopause, BP increases in women and reaches the same levels as in men [17,21]. Interestingly, females with hypertension tend to have less BP control than males despite higher treatment compliance [22]. These previous findings categorically underscore the relevance of understanding sex-specific differences in potential new hypertensive treatment strategies.

In this study, the BP of male SHRs was around 200 mmHg, which is clinically considered a hypertensive emergency that needs very aggressive BP-lowering therapy [23]. A lower ratio of type 1/2 receptors of angiotensin II has been found in the blood vessels and kidneys of female SHRs than in male SHRs [24]. This may be one of the reasons for the less severe hypertension in female SHRs. Fifteen weeks of treatment with beta-glucan resulted in a mean decrease in BP of approximately 20 mmHg in male SHRs but had no effect on female SHRs. As expected, treatment with hydrochlorothiazide alone was beneficial in lowering BP in male SHRs. Furthermore, the combination of both beta-glucan and hydrochlorothiazide also resulted in a significant lowering of BP in male SHRs. Unexpectedly, hydrochlorothiazide did not have a favorable effect on female SHRs. Additionally, the combination of beta-glucan and hydrochlorothiazide was not effective in lowering BP in female SHRs. It should be noted that the mechanisms responsible for BP variation between males and females are not fully understood. It is believed that androgens may a play a role in BP variation. Currently, there is no sex-specific drug therapy for hypertension, as anti-hypertensive medications are thought to have the same efficacy in both sexes [25]. However, it should be noted that most clinical trials have had an under representation of women, which limits a clear understanding of its sex-specific efficacy [26]. Hence, it may be possible that hydrochlorothiazide is less effective in controlling very high BP in female SHRs. It should be noted that females achieve better BP reduction when treated with betablockers and calcium channel blockers [27]. Thiazide diuretics are known to have dose-related BP lowering effects [28]. The less-pronounced anti-hypertensive effects of hydrochlorothiazide in female SHRs may be dose related. This is particularly noteworthy, as previous evidence has indicated that females are more often prescribed diuretics such as hydrochlorothiazide [29].

Even a slight lowering of 2 mmHg in mean diastolic BP has been established as an effective way to manage the risk of coronary disease and stroke by 6% and 15%, respectively [30]. A recent metanalysis of randomized clinical trials that examined the effects of oat, oat beta-glucan-rich extracts, and avenanthramides showed that oat supplementation reduced diastolic BP in obese hypercholesterolemic participants [31]. A previous clinical study also showed that a three-week incorporation of oat beta-glucan into bread (3 g/day) in the diet of patients with type 2 diabetes and high BP resulted in a significant lowering of systolic BP [32]. Another study reported that, in healthy men and women aged between 30–65 years, a high fiber diet with 7.3 g of beta-glucan led to a modest lowering of BP [33]. In light of our results and previous studies, although individual and combination treatments with beta-glucan and hydrochlorothiazide resulted in the lowering of BP in male SHRs, an additive effect was not observed (when compared to either treatment alone). Thus, it is worthwhile to pursue the role of beta-glucan in comparison to other anti-hypertensive medications and combinations thereof.

Our previous studies showed that LV wall hypertrophy was present in 20-week-old male SHRs based on echocardiographic findings, such as LV weight/tibia length ratio, IVS, and LVPW [15,34]. In this study, we did not observe an increase in IVS and LVPW in 20-week-old male and female SHRs, but there was a trend towards an increase in heart/tibia length in both groups of SHRs. We also did not observe any LV dilatation, as there was no increase in LVID in male or female SHRs. This finding is consistent with previous findings that male SHRs did not exhibit LV dilation, as evidenced by there being no alteration in LVID R [15,34]. Although no cardiac structural changes were observed in SHRs and SHRs treated with beta-glucan or hydrochlorothiazide, it was curious to note that SHRs treated with the combination exhibited an increase in ventricular wall thickening (IVS and LVPW) or hypertrophy at diastole. This observation indicates an undesirable effect of combination treatment on cardiac structural parameters in SHRs. Impaired diastolic function, such as the prolongation of IVRT, is a major cardiac abnormality due to hypertension. Diastolic dysfunctional abnormalities cause an increased risk of mortality in large number of heart failure patients [35]. Male and female SHRs treated with the vehicle showed evidence of diastolic dysfunction, such as the prolongation of IVRT, at 20 weeks. Prolonged IVRT represents an impaired relaxation pattern of LV and inadequate LV filling and has been reported as preceding the development of systolic dysfunction as well. A prolongation of IVRT is reported in hypertensive patients [36,37,38]. Anti-hypertensive treatment is known to improve filling properties and improve diastolic function [39,40]. In this study, hydrochlorothiazide and beta-glucan + hydrochlorothiazide treatments resulted in a reduction in IVRT in male SHRs, suggesting that these treatments were effective in preventing diastolic dysfunction. IVRT was not significantly lowered following any treatment in female SHRs, even though a clear trend towards a decrease was observed. This finding suggests that the treatments are less effective in improving diastolic function in female SHRs. Our findings also show that there was a reduction of LVEF in male and female SHRs treated with the vehicle at 20 weeks of age, representing the onset of systolic dysfunction. Treatment with hydrochlorothiazide or beta-glucan + hydrochlorothiazide improved LVEF in male and female SHRs, which suggests that the treatment was effective in preventing systolic dysfunction. The combination treatment also showed a trend towards further improvement. Diuretics are effective for reducing LV preload and afterload, which may help in improving LVEF [41]. 

It is important to note that both IVRT and LVEF in male and female SHRs treated with beta-glucan alone were not significantly improved, even though there was a clear trend towards improvement. These findings suggest differential effects of beta-glucan on the mechanisms underlying BP and heart function in SHRs. Other systolic functional parameters were largely unaffected in all groups, except for a significant decrease observed in CO and SV in male SHRs treated with the combination of beta-glucan + hydrochlorothiazide. The significance of this reduction is not clear given the significant improvement observed in LVEF in SHRs treated with the combination.

Oxidative stress is generally considered a major factor underlying the pathogenesis of cardiovascular disease, including hypertension [42,43]. The current study showed that male and female SHRs had significantly increased serum levels of MDA, a widely used marker of oxidative stress. Male SHRs also had significantly increased levels of MDA in their LVs and aortas. Our previous studies also reported that male SHRs exhibited higher levels of oxidative stress-related markers by 20 weeks of age [15,34]. Beta-glucan alone, hydrochlorothiazide alone, and the combination of both beta-glucan and hydrochlorothiazide were able to prevent the elevation of MDA in the serums of both male and female SHRs, as well as the LVs and aortas of male SHRs. Our previous study also showed that beta-glucan decreased the levels of malondialdehyde in male SHRs [44]. These results suggest that beta-glucan and hydrochlorothiazide are very effective in preventing oxidative stress in the setting of hypertension in SHRs. Antioxidant enzymes, such as SOD and catalase, are front-line antioxidant molecules that play a crucial role in maintaining redox status [43,45]. Serum levels of SOD activity in male SHRs were significantly lower at 20 weeks, whereas in female SHRs, SOD activity was not decreased. A previous study also reported that male SHRs have greater total body oxidative stress and a depressed plasma SOD activity compared with female SHRs [43]. According to another study, SOD protein expression is also lower in the myocardium of male SHRs compared with male WKY rats [46]. Male SHRs treated with beta-glucan or the combination of beta-glucan + hydrochlorothiazide had higher levels of SOD activity, suggesting an improved antioxidant status; hydrochlorothiazide treatment also led to a trend toward the improvement of SOD activity in male SHRs. Male and female SHRs and WKY rats had comparable levels of catalase. This suggests that catalase activity was not impaired in male and female SHRs at 20 weeks. It should also be noted that a previous study reported that SHRs have lower levels of catalase activity [47]. Further studies are needed to understand these discrepancies. However, it is curious to note that male SHRs treated with hydrochlorothiazide had significantly lower levels of catalase activity when compared to WKY rats and control SHRs, suggesting an adverse effect of hydrochlorothiazide treatment on this critical antioxidant in SHRs. 

Inflammation is also considered an aspect of pathological change associated with hypertension [48,49]. Male SHRs had elevated levels of a pro-inflammatory marker, TNF-alpha, in their serums at 20 weeks in this study; however, male SHRs treated with beta-glucan, hydrochlorothiazide, or the combination did not have elevated levels of TNF-alpha. Additionally, TNF-alpha was not elevated in the LVs of male SHRs. It is interesting to note that female SHRs did not have elevated levels of the pro-inflammatory marker, TNF-alpha, in their serums or LVs at 20 weeks. NO also plays an important role as a main modulator of vascular homeostasis. Increased oxidation may result in an uncoupling of NO synthase and in the production of deleterious peroxynitrate [50]. In this study, there was a decrease in the ratio of nitrate/nitrite (a measure of NO) in female SHRs, whereas male SHRs did not have a significantly lower NO, even though there was a trend towards a reduction. Thus, it appears that NO production was not impaired at this time point in male SHRs. This is consistent with one of our previous studies [44]. The renin-angiotensin-aldosterone system regulates BP, sodium retention, pressure natriuresis, salt sensitivity, vasoconstriction, endothelial dysfunction, and vascular injury. In this study, female SHRs had lower levels of angiotensin II compared to male SHRs. Beta-glucan, hydrochlorothiazide, and combination treatments were able to decrease the levels of angiotensin II in male SHRs. In female SHRs, only beta-glucan reduced the levels of angiotensin II, which suggests that hydrochlorothiazide and the combination treatment had sex-specific effects on angiotensin II production. SNS hyperactivity, such as increased levels of norepinephrine, is relevant to both the generation and maintenance of hypertension. In this study, norepinephrine was elevated in male and female SHRs. In male SHRs, treatment with beta-glucan led to a decrease in norepinephrine, whereas in female SHRs, treatment with beta-glucan, hydrochlorothiazide, or combination of both was able to prevent the increase in levels of norepinephrine. BP-lowering effects of the treatments in SHRs may be partly related to a decrease in the levels of angiotensin II and norepinephrine. Hydroxy proline was not significantly different between male and female WKY rats and male and female SHRs, suggesting there was no increase in cardiac fibrosis at this time point. 

Oat-derived ingredients are considered to have health value for humans as they may result in a lowering of the risk of chronic diseases, such as dyslipidemia, hyperglycemia, and hypertension [44]. The dose of beta-glucan (305 mg per kg body weight) used in our animal study is equivalent to the recommended daily intake of beta-glucan (3–4 g per day), which reduces the risk of dyslipidemia [44] in humans. A daily intake of 100 g of rolled oats is suggested to attain 3–4 g of beta-glucan per day via diet [10]. Therefore, our findings suggest that the current recommended daily intake of beta-glucan may also result in BP lowering in humans with hypertension.

## 5. Conclusions

We conclude that a 15-week treatment with oat beta-glucan alone and the combination of oat beta-glucan with hydrochlorothiazide are beneficial in lowering high BP in male SHRs but not in female SHRs. Beta-glucan + hydrochlorothiazide treatment also improved systolic and diastolic function in male and female SHRs. Further studies are warranted to understand the sex-specific efficacy of beta-glucan for the management of hypertension and cardiac dysfunction. Furthermore, well-designed human trials are also needed to establish the clinical benefits of oat beta-glucan in similar populations.

## Data Availability

Available on reasonable request.

## References

[1] NCD Risk Factor Collaboration (NCD-RisC) (2017). Worldwide trends in blood pressure from 1975 to 2015: A pooled analysis of 1479 population-based measurement studies with 19.1 million participants. Lancet.

[2] Salem H., Hasan D.M., Eameash A., El-Mageed H.A., Hasan S., Ali R. (2018). Worldwide prevalence of hypertension: A pooled meta-analysis of 1670 studies in 71 countries with 29.5 million participants. J. Am. Coll. Cardiol..

[3] Oparil S., Acelajado M.C., Bakris G.L., Berlowitz D.R., Cifkova R., Dominiczak A.F., Grassi G., Jordan J., Poulter N.R., Rodgers A. (2018). Hypertension. Nat. Rev. Dis. Primers.

[4] Ekstrom M., Hellman A., Hasselstrom J., Hage C., Kahan T., Ugander M., Wallen H., Persson H., Linde C. (2020). The transition from hypertension to hypertensive heart disease and heart failure: The PREFERS Hypertension study. ESC Heart Fail..

[5] Chen Y.F. (1996). Sexual dimorphism of hypertension. Curr. Opin. Nephrol. Hypertens..

[6] Lattimer J.M., Haub M.D. (2010). Effects of dietary fiber and its components on metabolic health. Nutrients.

[7] Brown L., Rosner B., Willett W.W., Sacks F.M. (1999). Cholesterol-lowering effects of dietary fiber: A meta-analysis. Am. J. Clin. Nutr..

[8] Whitehead A., Beck E.J., Tosh S., Wolever T.M. (2014). Cholesterol-lowering effects of oat beta-glucan: A meta-analysis of randomized controlled trials. Am. J. Clin. Nutr..

[9] Ho H.V., Sievenpiper J.L., Zurbau A., Blanco Mejia S., Jovanovski E., Au-Yeung F., Jenkins A.L., Vuksan V. (2016). A systematic review and meta-analysis of randomized controlled trials of the effect of barley beta-glucan on LDL-C, non-HDL-C and apoB for cardiovascular disease risk reduction(i–iv). Eur. J. Clin. Nutr..

[10] Maki K.C., Galant R., Samuel P., Tesser J., Witchger M.S., Ribaya-Mercado J.D., Blumberg J.B., Geohas J. (2007). Effects of consuming foods containing oat beta-glucan on blood pressure, carbohydrate metabolism and biomarkers of oxidative stress in men and women with elevated blood pressure. Eur. J. Clin. Nutr..

[11] Marques F.Z., Nelson E., Chu P.Y., Horlock D., Fiedler A., Ziemann M., Tan J.K., Kuruppu S., Rajapakse N.W., El-Osta A. (2017). High-Fiber Diet and Acetate Supplementation Change the Gut Microbiota and Prevent the Development of Hypertension and Heart Failure in Hypertensive Mice. Circulation.

[12] Bing O.H., Conrad C.H., Boluyt M.O., Robinson K.G., Brooks W.W. (2002). Studies of prevention, treatment and mechanisms of heart failure in the aging spontaneously hypertensive rat. Heart Fail. Rev..

[13] Doggrell S.A., Brown L. (1998). Rat models of hypertension, cardiac hypertrophy and failure. Cardiovasc. Res..

[14] Juric D., Wojciechowski P., Das D.K., Netticadan T. (2007). Prevention of concentric hypertrophy and diastolic impairment in aortic-banded rats treated with resveratrol. Am. J. Physiol. Heart Circ. Physiol..

[15] Aloud B.M., Raj P., McCallum J., Kirby C., Louis X.L., Jahan F., Yu L., Hiebert B., Duhamel T.A., Wigle J.T. (2018). Cyanidin 3-O-glucoside prevents the development of maladaptive cardiac hypertrophy and diastolic heart dysfunction in 20-week-old spontaneously hypertensive rats. Food Funct..

[16] Zheng X., Qiu Y., Zhong W., Baxter S., Su M., Li Q., Xie G., Ore B.M., Qiao S., Spencer M.D. (2013). A targeted metabolomic protocol for short-chain fatty acids and branched-chain amino acids. Metabolomics.

[17] Ahmed S., Hu R., Leete J., Layton A.T. (2019). Understanding sex differences in long-term blood pressure regulation: Insights from experimental studies and computational modeling. Am. J. Physiol. Heart Circ. Physiol..

[18] Olivera S., Graham D. (2022). Sex differences in preclinical models of hypertension. J. Hum. Hypertens..

[19] Ouchi Y., Share L., Crofton J.T., Iitake K., Brooks D.P. (1987). Sex difference in the development of deoxycorticosterone-salt hypertension in the rat. Hypertension.

[20] Wiinberg N., Hoegholm A., Christensen H.R., Bang L.E., Mikkelsen K.L., Nielsen P.E., Svendsen T.L., Kampmann J.P., Madsen N.H., Bentzon M.W. (1995). 24-h ambulatory blood pressure in 352 normal Danish subjects, related to age and gender. Am. J. Hypertens..

[21] Samargandy S., Matthews K.A., Brooks M.M., Barinas-Mitchell E., Magnani J.W., Thurston R.C., El Khoudary S.R. (2022). Trajectories of Blood Pressure in Midlife Women: Does Menopause Matter?. Circ. Res..

[22] Gu Q., Burt V.L., Paulose-Ram R., Dillon C.F. (2008). Gender differences in hypertension treatment, drug utilization patterns, and blood pressure control among US adults with hypertension: Data from the National Health and Nutrition Examination Survey 1999–2004. Am. J. Hypertens..

[23] Lagi A., Cencetti S. (2015). Hypertensive emergencies: A new clinical approach. Clin. Hypertens..

[24] Silva-Antonialli M.M., Tostes R.C., Fernandes L., Fior-Chadi D.R., Akamine E.H., Carvalho M.H., Fortes Z.B., Nigro D. (2004). A lower ratio of AT1/AT2 receptors of angiotensin II is found in female than in male spontaneously hypertensive rats. Cardiovasc. Res..

[25] Wright J.T., Whelton P.K., Johnson K.C., Snyder J.K., Reboussin D.M., Cushman W.C., Williamson J.D., Pajewski N.M., Cheung A.K., Lewis C.E. (2021). SPRINT Revisited: Updated Results and Implications. Hypertension.

[26] Kim E.S.H., Menon V. (2009). Status of Women in Cardiovascular Clinical Trials. Arterioscler. Thromb. Vasc. Biol..

[27] Tamargo J., Rosano G., Walther T., Duarte J., Niessner A., Kaski J.C., Ceconi C., Drexel H., Kjeldsen K., Savarese G. (2017). Gender differences in the effects of cardiovascular drugs. Eur. Heart J. Cardiovasc. Pharmacother..

[28] Musini V.M., Nazer M., Bassett K., Wright J.M. (2014). Blood pressure-lowering efficacy of monotherapy with thiazide diuretics for primary hypertension. Cochrane Database Syst. Rev..

[29] Gerdts E., Sudano I., Brouwers S., Borghi C., Bruno R.M., Ceconi C., Cornelissen V., Dievart F., Ferrini M., Kahan T. (2022). Sex differences in arterial hypertension. Eur. Heart J..

[30] Bazzano L.A., Green T., Harrison T.N., Reynolds K. (2013). Dietary approaches to prevent hypertension. Curr. Hypertens. Rep..

[31] Llanaj E., Dejanovic G.M., Valido E., Bano A., Gamba M., Kastrati L., Minder B., Stojic S., Voortman T., Marques-Vidal P. (2022). Effect of oat supplementation interventions on cardiovascular disease risk markers: A systematic review and meta-analysis of randomized controlled trials. Eur. J. Nutr..

[32] Liatis S., Tsapogas P., Chala E., Dimosthenopoulos C., Kyriakopoulos K., Kapantais E., Katsilambros N. (2009). The consumption of bread enriched with betaglucan reduces LDL-cholesterol and improves insulin resistance in patients with type 2 diabetes. Diabetes Metab..

[33] He J., Streiffer R.H., Muntner P., Krousel-Wood M.A., Whelton P.K. (2004). Effect of dietary fiber intake on blood pressure: A randomized, double-blind, placebo-controlled trial. J. Hypertens..

[34] Thandapilly S.J., Wojciechowski P., Behbahani J., Louis X.L., Yu L., Juric D., Kopilas M.A., Anderson H.D., Netticadan T. (2010). Resveratrol prevents the development of pathological cardiac hypertrophy and contractile dysfunction in the SHR without lowering blood pressure. Am. J. Hypertens..

[35] Aziz F., Tk L.A., Enweluzo C., Dutta S., Zaeem M. (2013). Diastolic heart failure: A concise review. J. Clin. Med. Res..

[36] Mureddu G.F., de Simone G., Greco R., Rosato G.F., Contaldo F. (1997). Left ventricular filling in arterial hypertension. Influence of obesity and hemodynamic and structural confounders. Hypertension.

[37] de Simone G., Greco R., Mureddu G., Romano C., Guida R., Celentano A., Contaldo F. (2000). Relation of left ventricular diastolic properties to systolic function in arterial hypertension. Circulation.

[38] Mattioli A.V., Zennaro M., Bonatti S., Bonetti L., Mattioli G. (2004). Regression of left ventricular hypertrophy and improvement of diastolic function in hypertensive patients treated with telmisartan. Int. J. Cardiol..

[39] Wachtell K., Bella J.N., Rokkedal J., Palmieri V., Papademetriou V., Dahlof B., Aalto T., Gerdts E., Devereux R.B. (2002). Change in diastolic left ventricular filling after one year of antihypertensive treatment: The Losartan Intervention For Endpoint Reduction in Hypertension (LIFE) Study. Circulation.

[40] Muller-Brunotte R., Kahan T., Malmqvist K., Ring M., Edner M. (2006). Tissue velocity echocardiography shows early improvement in diastolic function with irbesartan and atenolol therapy in patients with hypertensive left ventricular hypertrophy. Results form the Swedish Irbesartan Left Ventricular Hypertrophy Investigation vs Atenolol (SILVHIA). Am. J. Hypertens..

[41] Ito H., Ishii K., Kihara H., Kasayuki N., Nakamura F., Shimada K., Fukuda S., Iwakura K., Yoshikawa J. (2012). Adding thiazide to a renin-angiotensin blocker improves left ventricular relaxation and improves heart failure in patients with hypertension. Hypertens. Res..

[42] Sullivan J.C., Sasser J.M., Pollock J.S. (2007). Sexual dimorphism in oxidant status in spontaneously hypertensive rats. Am. J. Physiol. Regul. Integr. Comp. Physiol..

[43] Khullar M., Relan V., Sehrawat B.S. (2004). Antioxidant activities and oxidative stress byproducts in human hypertension. Hypertension.

[44] Raj P., Ames N., Joseph Thandapilly S., Yu L., Netticadan T. (2020). The effects of oat ingredients on blood pressure in spontaneously hypertensive rats. J. Food Biochem..

[45] Agarwal D., Haque M., Sriramula S., Mariappan N., Pariaut R., Francis J. (2009). Role of proinflammatory cytokines and redox homeostasis in exercise-induced delayed progression of hypertension in spontaneously hypertensive rats. Hypertension.

[46] Adler S., Huang H. (2004). Oxidant stress in kidneys of spontaneously hypertensive rats involves both oxidase overexpression and loss of extracellular superoxide dismutase. Am. J. Physiol. Renal Physiol..

[47] Chan S.H., Tai M.H., Li C.Y., Chan J.Y. (2006). Reduction in molecular synthesis or enzyme activity of superoxide dismutases and catalase contributes to oxidative stress and neurogenic hypertension in spontaneously hypertensive rats. Free Radic. Biol. Med..

[48] Sanz-Rosa D., Oubiña M.P., Cediel E., Heras N.d.l., Vegazo O., Jiménez J., Lahera V., Cachofeiro V. (2005). Effect of AT1 receptor antagonism on vascular and circulating inflammatory mediators in SHR: Role of NF-κB/IκB system. Am. J. Physiol.-Heart Circ. Physiol..

[49] Patrick D.M., Van Beusecum J.P., Kirabo A. (2021). The role of inflammation in hypertension: Novel concepts. Curr. Opin. Physiol..

[50] Forstermann U., Xia N., Li H. (2017). Roles of Vascular Oxidative Stress and Nitric Oxide in the Pathogenesis of Atherosclerosis. Circ. Res..

